# Angiopoietin-1: an early biomarker of diabetic nephropathy?

**DOI:** 10.1186/s12967-021-03105-9

**Published:** 2021-10-13

**Authors:** Alexandra E. Butler, Ahmed Al-Qaissi, Thozhukat Sathyapalan, Stephen L. Atkin

**Affiliations:** 1 Department of Research, Royal College of Surgeons of Ireland, PO Box 15503, Adliya, Bahrain; 2 Academic Endocrinology, Diabetes and Metabolism, Hull York Medical School, Hull, UK; 3 Leeds Medical School, Leeds, UK

**Keywords:** Type 2 diabetes, Diabetic kidney disease, Biomarkers, Proteomics

Letter to the Editor

Diabetic kidney disease (DKD) with progression to end-stage renal disease (ESRD) is a much-feared diabetes complication. Early recognition is key to preventing decline in renal function and, hence, biomarkers to stratify risk of functional decline have been actively sought. In a recent publication, plasma proteomic analysis was performed using the SOMASCAN platform in two longitudinal exploratory studies of type 1 (T1D) and type 2 diabetes (T2D) patients with chronic kidney disease (CKD) stage-3 to identify candidate protective biomarkers against progressive renal function decline/progression to ESRD, findings validated in a T1D patient cohort with normal renal function [1]. Their findings distilled down to three proteins that showed a strong, additive protective effect against decline in renal function: angiopoeitin-1 (ANGPT1), tumor necrosis factor receptor superfamily 12 (TNFRSF12) and fibroblast growth factor 20 (FGF20) [1].

Glucose variability, potentiating hyper- or hypoglycemia, positively associates with both micro- and macrovascular diabetes complications [2] and may be an independent risk factor, seen by glucose variability accelerating renal injury in rats [3]. Stringent therapeutic regimens aimed at maintaining normoglycemia in diabetes patients have increase the frequency of hypoglycemic episodes [4].

We hypothesized that the protective biomarkers of renal function identified in the Joslin study [1] may be elevated early in T2D and be affected by glucose excursions including a hypoglycemic insult.

We performed a case-controlled study in 23 T2D and 23 control age-matched Caucasian subjects. Following a 10-h fast, each subject underwent a hyperinsulinemic clamp, as previously described [5]. Baseline plasma glucose in the T2D cohort was 7.6 ± 0.4 mmol/L (136.8 ± 7.2 mg/dl); glucose was normalized to 4.5 ± 0.1 mmol/L (81 ± 1.2 mg/dl) for 1-h. In the control cohort, plasma glucose was maintained at the baseline level of 4.9 ± 0.1 mmol/L (88.2 ± 1.8 mg/dl) during this normalization period. Thereafter, glucose was lowered to hypoglycemic levels, where blood glucose (BG) levels were 2.0 ± 0.03 mmol/L in T2D and 1.8 ± 0.05 mmol/L in controls. Plasma samples were collected at baseline, at glucose normalization (T2D only), at hypoglycemia and during the post-hypoglycemia follow up period (0.5, 1, 2, 4 and 24-h), as previously described [5].

Using the same Slow Off-rate Modified Aptamer (SOMA)-scan measurement (SOMAscan) proteomics platform as used by Dom et al. [1], we determined levels of the three validated renal protective proteins: ANGPT1, TNFSF12 and FGF20 at these timepoints.

The T2D cohort studied here had a relatively short disease duration (4.5 ± 2.2 years) with no diabetic complications and a normal eGFR, though BMI was increased versus controls (p = 0.001).

Protein levels throughout the study timecourse are shown in Fig. 1. At baseline, plasma FGF20 was higher in controls (p < 0.01), ANGPT1 was higher in T2D (p < 0.05) whilst there was a trend to lower TNFSF12 levels in T2D (Fig. 1A–C).

**Fig. 1:**
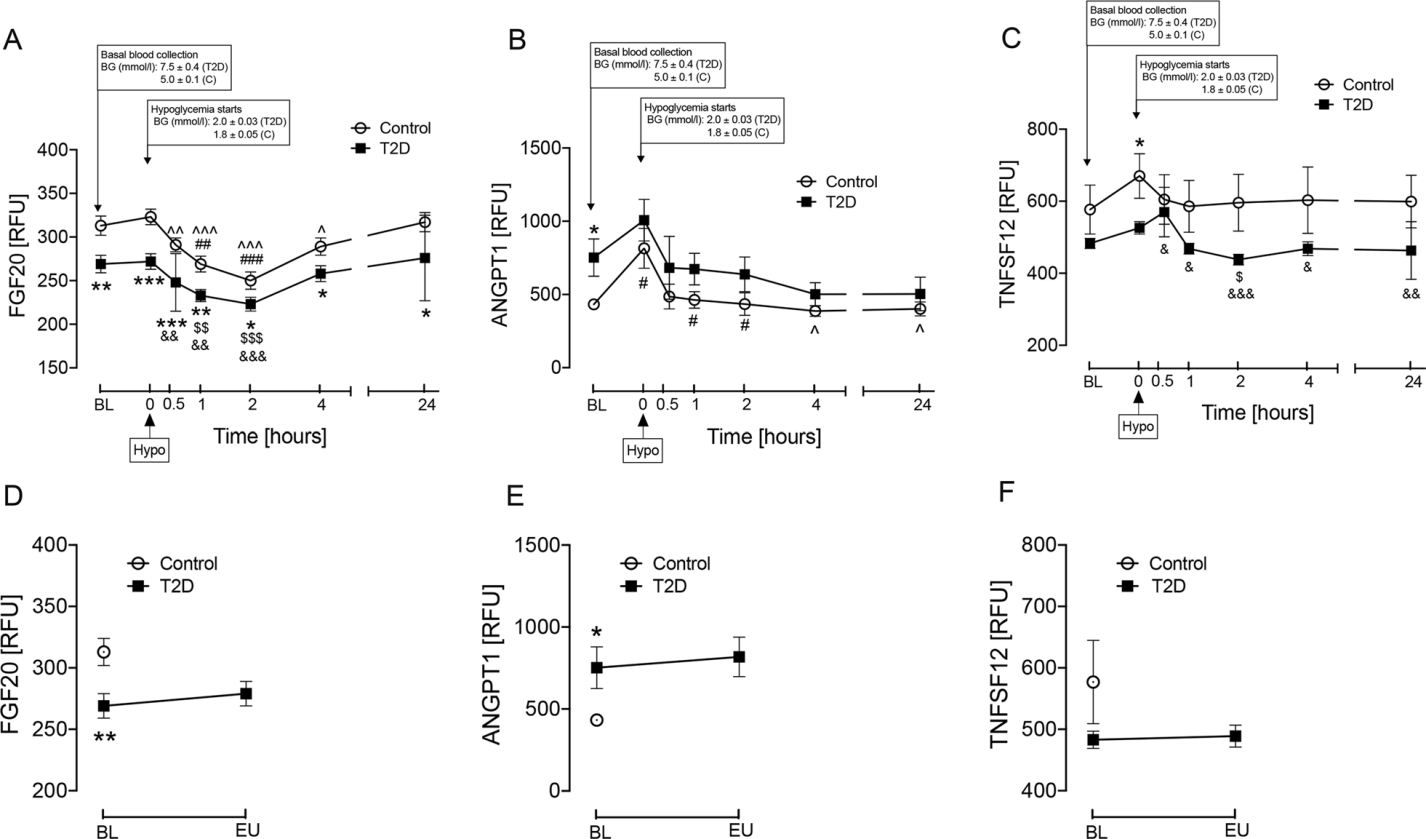
Circulatory levels of proteins protective of renal function at baseline, at hypoglycemia and at post-hypoglycemia timepoints in T2D and control subjects, and upon glucose level normalization in T2D subjects. Proteomic (Somalogic) analysis of fibroblast growth factor 20 [FGF20], angiopoietin-1 [ANGPT1] and tumor necrosis factor ligand superfamily member 12 [TNFSF12] was undertaken. **A**–**C** Blood sampling was performed at baseline (BL), at hypoglycemia (0 min) and post-hypoglycemia (30-min, 1-h, 2-h, 4-h and 24-h) for controls (white circles) and for T2D (black squares). At baseline (BL), blood glucose (BG) was 7.5 ± 0.4 mmol/L (for T2D) and 5.0 ± 0.1 mmol/L (for control, C). At point of hypoglycemia, blood glucose (BG) was 2.0 ± 0.03 mmol/L (for T2D) and 1.8 ± 0.05 mmol/L (for control). **D**–**F** Blood sampling was performed at baseline (BL) in both controls (white circles) and T2D (black squares), and at glucose normalization (BM) in T2D subjects [4.5 ± 0.1 mmol/L (81 ± 1.2 mg/dl)] for a duration of 1-h. In the control cohort, mean plasma glucose was maintained at the baseline level of 4.9 ± 0.1 mmol/L (88.2 ± 1.8 mg/dl) during this normalization period. Statistics: T2D vs control: *p < 0.05, **p < 0.01, ***p < 0.001; T2D BL vs subsequent timepoints: ^$^p < 0.05, ^$$^p < 0.01, ^$$$^p < 0.001; T2D hypoglycemia vs subsequent timepoints: ^&^p < 0.05, ^&&^p < 0.01, ^&&&^p < 0.001; Control BL vs subsequent timepoints: ^#^p < 0.05; ^##^p < 0.01, ^###^p < 0.001; Control hypoglycemia vs subsequent timepoints: ^p < 0.05. ^^p < 0.01, ^^^p < 0.001. RFU: relative fluorescent unit

At euglycemia in T2D there was no difference in FGF20, ANGPT1 or TNFSF12 (Fig. 1D–F).

At hypoglycemia, FGF20 remained higher in controls (p < 0.001); ANGPT1 levels increased more in controls than T2D, achieving similar levels in both cohorts (p = ns). Likewise, levels of TNFSF12 increased more in controls than in T2D, resulting in a significant difference between cohorts (p < 0.05).

During the post-hypoglycemia follow-up period, FGF20 remained consistently higher in controls (30-min and 2-h: p < 0.001; 1-h: p < 0.01; 4- and 24-h, p < 0.05). ANGPT1 and TNFSF12 levels were not different between cohorts at any post-hypoglycemia timepoint.

Interestingly, when glucose levels were normalized in the T2D cohort (Fig. 1D–F), protein levels were unchanged, indicating that glucose variability did not affect the circulating protein levels.

In early diabetes with normal eGFR, plasma ANGPT1 is elevated, suggesting that this may be an early marker of renal dysfunction, whilst TNFSF12 and FGF20 are not. This may indicate that ANGPT1 is an early biomarker and, upon diagnosis, should be measured and, if elevated, additional care taken to aggressively prevent renal disease. Post-diagnosis, should a subsequent increase in TNFSF12 and FGF20 occur, this may indicate progression of renal disease, even without overt changes in eGFR or an increased albumin:creatinine ratio, and would again justify aggressive preventative measures. Further, our results indicate that levels of ANGPT1, TNFSF12 and FGF20 are unaffected by glucose normalization in T2D and therefore not prone to variability in response to glucose fluctuations; therefore, fasting samples are not required.

Strengths of this study are use of the identical SOMAscan proteomics platform as in the reference paper [1], making our results directly comparable; the enrollment of a population of T2D with short duration of disease and without diabetes complications, who had a normal GFR and who were treatment naïve. Limitations include relatively small study numbers and that this was a homogeneous Caucasian population, so the results may not be applicable to other ethnic groups.

In conclusion, ANGPT1 may be the earliest biomarker of potential diabetes-related renal disease progression. This data suggests that prospective studies be undertaken to confirm or refute whether this reflects future clinical risk and would therefore impact clinical practice.

ClinicalTrials.gov NCT03102801. Date of registration April 6, 2017, retrospectively registered. https://clinicaltrials.gov/ct2/show/NCT03102801?term=NCT03102801&draw=2&rank=1

## Data Availability

All the data for this study will be made available upon reasonable request to the corresponding author.
